# Home and Community Based Services for People With Dementia

**DOI:** 10.1093/geroni/igaa057.2316

**Published:** 2020-12-16

**Authors:** Howard Degenholtz, Raymond Van Cleve

**Affiliations:** 1 University of Pittsburgh, Pittsburgh, Pennsylvania, United States; 2 U.S. Department of Veterans Affairs, West Haven, Connecticut, United States

## Abstract

Home and Community Based Services has grown as an alternative to nursing homes over the past 30 years. While there are extensive data on nursing home staffing, there is a dearth of similar information about Medicaid financed home care. We use data from 2014-2016 Pennsylvania Medicaid to examine personal care for elderly with and without dementia across the range of physical disability. One challenge is that even though physical function can be measured in terms of discrete tasks (i.e., limitation in bathing, dressing, toileting), analysis of the amount of care people receive has to take into account the combination of dementia and combinations of ADL limitations. We found that older adults with dementia receive one hour more of personal care per day at the lowest level of disability and 1.5 hours at the highest level. The increased need for caregiving hours should be incorporated into policies that guide HCBS programs.

